# Undiagnosed Patent Foramen Ovale Presenting as Retinal Artery Occlusion—An Emerging Association

**DOI:** 10.1155/2009/248269

**Published:** 2009-11-22

**Authors:** Hiten G. Sheth, Tania Laverde-Konig, Jyoti Raina

**Affiliations:** Department of Ophthalmology, North Middlesex University Hospital, Sterling Way, London N18 1QX, UK

## Abstract

*Purpose*. To report patent foramen ovale (PFO) as the cause of retinal artery occlusion in a young and previously fit male and discuss the appropriate medical and surgical management options. *Methods*. Interventional case report with serial fundus photographs of an 18-year-old male presenting to the eye casualty with sudden onset left visual loss. *Results*. Visual acuities were 6/24 left and 6/4 right with a left afferent pupillary defect. Slitlamp examination confirmed a left hemiretinal artery occlusion and subsequent cardiology review with transoesophageal echocardiography revealed patent foramen ovale which was closed surgically. *Conclusions*. PFO is not uncommon and is often covert but predisposes individuals to embolic events. These events may be ophthalmic with visual sequelae and so ophthalmologists, physicians, and other healthcare personnel should be aware of this important and emerging association.

## 1. Introduction

Patent foramen ovale (PFO) represents a persistent congenital interatrial communication of the heart, which failed to close normally within the first year of life, thus creating the potential for a right-to-left shunt. The prevalence of PFO is reported as 10–15% of the population by contrast transthoracic echocardiography and 26% in autopsy studies. Although generally not symptomatic, PFO predisposes the individual to embolic events which may involve the eye.

## 2. Case Report

**Figure 1:**
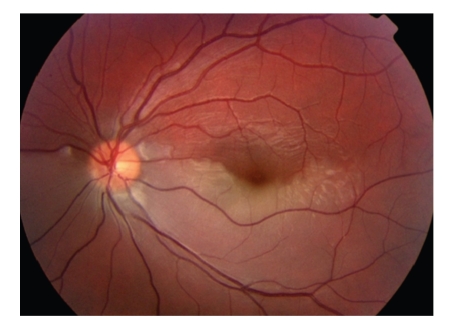
Colour photograph of left fundus at presentation, showing acute left hemiretinal artery occlusion and associated retinal oedema.

An 18-year-old male presented to the eye casualty with a history of left visual loss, which had occurred suddenly an hour previously. He had no past ophthalmic history, gave a past medical history of classical migraine (with visual aura and headache), and had endured a long distance bus journey the preceding day. There was no history of cigarette smoking or illicit drug use. Visual acuities were 6/24 left unaided not improving with pinhole and 6/4 unaided right. A left afferent pupillary defect (RAPD) was present, and loss of left superior visual field to confrontation with red pin was demonstrated. Intraocular pressures were normal and gonioscopy confirmed open iridocorneal angles. Dilated fundal examination revealed a left inferior hemi-retinal artery occlusion (Figure 1). Systemic neurological examination was normal, and blood pressure was 115/68. Immediate treatment included aspirin orally, acetazolomide intravenously, ocular massage, and rebreathing into a paper bag. 

**Figure 2:**
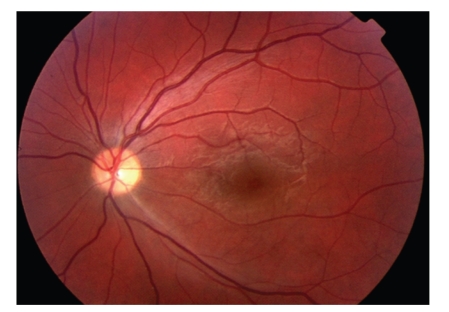
Colour photograph of left fundus 1 month after initial artery occlusion showing marked resolution in signs and sclerosis of vessel.

At 3-day review, left visual acuity had improved to 6/6 with a still persistent RAPD. Full blood count, urea and electrolytes, clotting, inflammatory markers, and thrombophilia screen were within normal range. He was reviewed in cardiology, and right-to-left shunting across a patent foramen ovale was revealed with transoesophageal echocardiography. Magnetic resonance imaging of the brain revealed discrete abnormalities consistent with the previous localised ischaemic events from emboli. He was treated with both aspirin and clopidogrel and at 1-month review, visual acuity remained good at 6/6, the retinal oedema had resolved and a sclerosed branch retinal vessel was evident (Figure 2). He subsequently underwent successful percutaneous closure of the cardiac lesion and 1 year on from initial presentation he remains free of visual or other neurological symptoms with an acuity of 6/6.

## 3. Discussion and Conclusion

Retinal artery occlusion in young patients is rare and only a handful of other cases with PFO as the cause are reported [1–4]. PFO is more common than perhaps appreciated by the medical profession, with an average prevalence at autopsy of 26% [5]. Most patients with an incidental PFO and no other systemic problems generally receive no treatment. When associated with otherwise unexplained ophthalmic or neurologic events, treatment may be medical (to include aspirin, clopidogrel, or warfarin) or surgical (in the form of a catheter-based procedure using Dacron patches or percutaneous surgical closure with primary closure with sutures). The potential complications of surgery must be carefully balanced against the risk of recurrent cerebral or ophthalmic embolic events. Interestingly, our patient had suffered classical migraine attacks for 5 years prior to his retinal artery occlusion, and there has been much recent discussion implicating PFO in the aetiology of migraine headache [6] and advocating surgical closure as a safe and effective treatment for migraine [7].

Ophthalmologists should thus consider PFO in patients diagnosed with retinal artery occlusion, particularly younger patients in whom no other aetiological risk factors can be identified, and asking about a history of migraine attacks may also be helpful in reaching the final diagnosis. PFO with ophthalmic and visual sequelae is an important emerging association and prompt diagnosis and liaison with cardiologists will help avoid unnecessary future ocular or nonocular embolic events and associated morbidity.
